# Lactic Acid Bacteria-Mediated Fermentation Drives Metabolic Remodeling of *Centella Asiatica* (L.) Urb. toward Acidic Triterpenoids with Neuroinflammation-Related Bioactivity

**DOI:** 10.1021/acsomega.6c00935

**Published:** 2026-05-04

**Authors:** Da Hye Ryu, Jwa Yeong Cho, Jae Woon Jung, Hyeong Ho Cha, Hye Min Kim, Na-Yun Park, Na-Hyun Ahn, Joo-Hee Lee, Jong Soon Park, Deuk-Sik Lee, Seung-Hoon Yang, Ho-Youn Kim

**Affiliations:** † Smart Farm Research Center, 58975 Korea Institute of Science and Technology (KIST), Gangneung 25451, Republic of Korea; ‡ Department of Biomedical Engineering, College of Life Science and Biotechnology, 34942 Dongguk University, Seoul 04620, Republic of Korea; § Natural Product Applied Science, KIST School, University of Science and Technology (UST), Gangneung, Gangwon 25451, Republic of Korea; ∥ Life Science Institute, Well-being LS Ltd., Gangneung, Gangwon 25451, Republic of Korea

## Abstract

*Centella asiatica* Urb. is a medicinal plant rich in triterpenoid constituents with a reported neurobiological relevance. Its major metabolites are glycosylated triterpenoids, such as asiaticoside and madecassoside, whereas the corresponding acidic triterpenoids, including asiatic acid and madecassic acid, are typically present at low abundance. Given the increasing interest in how metabolic forms of natural products influence biological activity, this study investigated whether lactic acid bacteria (LAB)-mediated fermentation could induce metabolic remodeling of *C. asiatica* through microbial biotransformation. LAB fermentation markedly altered the secondary metabolite profile, which was characterized by a reduction in phenolic compounds and glycosylated triterpenoids and a pronounced enrichment of acidic triterpenoids. This compositional shift was accompanied by changes in bioactivity, including a decreased antioxidant capacity but enhanced anti-inflammatory effects in macrophage cells. Fermented *C. asiatica* significantly suppressed the expression of proinflammatory cytokines (TNF-α and IL-1β) and modulated amyloid-β and tau protein aggregation behavior in vitro. Furthermore, in an Alzheimer’s disease transgenic mouse model, fermented extracts were associated with reduced amyloid plaque deposition, as assessed by Thioflavin S staining. Collectively, these results demonstrate that LAB-mediated fermentation drives functional metabolic remodeling of *C. asiatica* by altering the triterpenoid composition and bioactivity profiles. This work highlights microbial biotransformation as a versatile strategy for modulating the biological attributes of plant-derived natural products and for exploring relationships between chemical form and bioactivity in complex biological systems.

## Graphical abstract

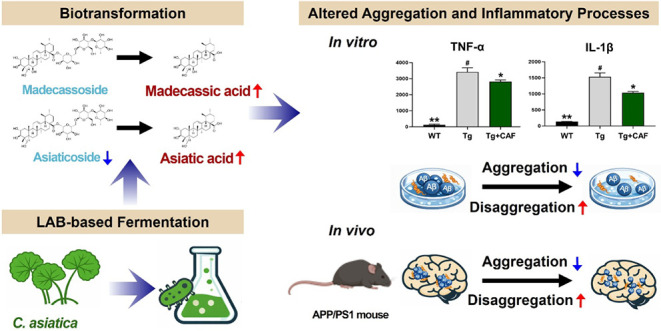

## 1 Introduction

The global aging of the population has led to a steady increase in age-associated disorders, among which Alzheimer’s disease (AD) represents one of the most prevalent neurodegenerative conditions.^1^ AD accounts for approximately 60–80% of dementia cases and is characterized by progressive neuronal dysfunction and cognitive decline.^2,3^ With aging, the capacity to regulate intracellular reactive oxygen species (ROS) declines, and excessive oxidative stress has been implicated in the onset of various neurodegenerative disorders, including AD.^4^ Elevated ROS levels impair microglial function and induce tau protein hyperphosphorylation through dysregulation of kinase and phosphatase activities, thereby promoting the formation of neurofibrillary tangles.^5,6^ Similarly, disruption of the balance between amyloid-β (Aβ) production and clearance results in Aβ accumulation, contributing to neuronal loss and brain atrophy.^7,8^ Consequently, extensive research has focused on elucidating the pathological role of oxidative stress in AD progression.^4^

However, accumulating evidence suggests that antioxidant capacity alone does not fully explain the complexity of AD pathology. While oxidative stress plays a critical role during the early stages of the disease, sustained neuroinflammation and protein aggregation become dominant pathological drivers as AD advances.^9^ Accordingly, strategies that modulate inflammatory signaling and amyloidogenic processes are increasingly recognized as important components in approaches addressing AD-related pathology.^10^

Given the progressive and irreversible nature of AD, there has been growing interest in understanding the biological properties of natural products in relation to AD-associated pathological processes.^11^ From a histological perspective, various natural substances have been reported to influence AD-associated processes.^6,8^ In parallel with the increasing prevalence of AD, research interest in plant-derived materials with biological relevance to neurodegenerative pathology has expanded.^2,3^ Previous studies have reported that natural antioxidants, including phenolics (polyphenols; a broad class of plant secondary metabolites characterized by one or more phenolic rings) and flavonoids (a major subclass of phenolics), can interfere with Aβ and tau aggregation by inhibiting their formation or promoting disaggregation as well as by chelating metal ions such as Cu^2+^, Zn^2+^, and Fe^2+3^.

*Centella asiatica* (L.) Urb. has been reported to exhibit biological activities relevant to the central nervous system, including effects on phospholipase-related pathways and amyloid-associated processes.^1,12^ Typically, saponins constitute approximately 1–8% of *C. asiatica* plant materials.^13^ A substantial proportion of these saponins comprises triterpene saponins and sapogenins derived from ursane- or oleanane-type skeletons, including asiaticoside (AS) and madecassoside (MS) as major triterpene saponins, and asiatic acid (AA) and madecassic acid (MA) as their corresponding triterpene sapogenins (aglycones), collectively referred to as centellosides.^14^ These triterpenoids exhibit distinct biological activities depending on their structural forms.^15,16^ Especially in ursane- or oleanane-type triterpenoids, the hydroxyl group at the C-3 position and the carboxyl group at the C-28 position play important roles in determining molecular polarity and interactions with biomolecules, and modifications at these sites have frequently been reported to be associated with changes in bioactivity. *C. asiatica* predominantly contains glycosylated triterpenoids (AS and MS), whereas the corresponding acidic triterpenoids (AA, MA) are present only at trace levels in the native plant (Figure 1).^17^

**Figure 1:**
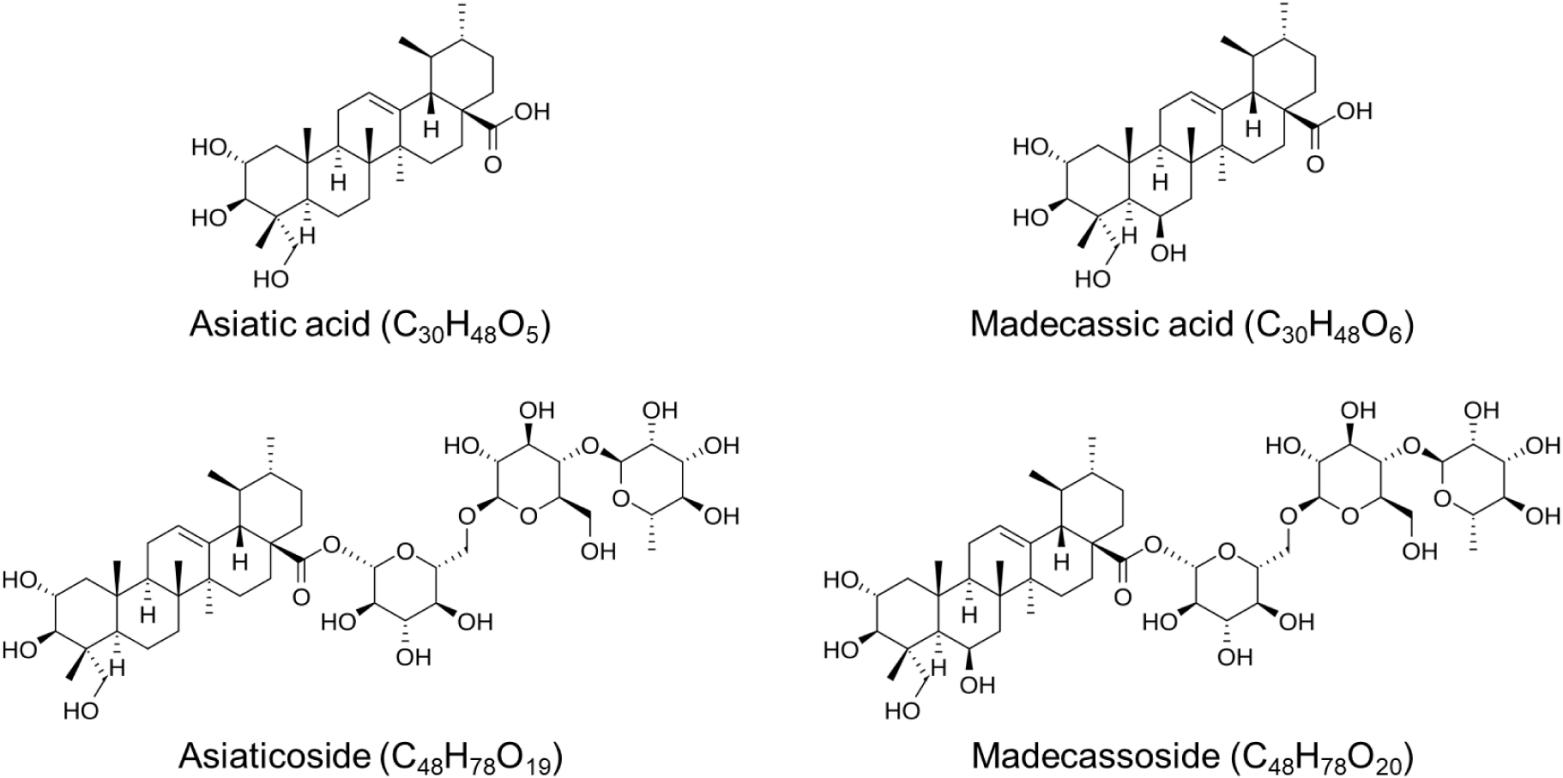
Chemical structures of asiaticoside (AS), madecassoside (MS), asiatic acid (AA), and madecassic acid (MA).

Although these triterpenoids are considered the principal contributors to the neuroactivity of *C. asiatica*, cellular and molecular mechanisms of action differ substantially according to the specific triterpenoid form.^18^ Notably, acidic triterpenoids (AA and MA) represent the biologically active aglycone forms, and accumulating evidence indicates that glycosylated triterpenoids require metabolic conversion to their acidic counterparts in vivo to exert their full biological activity. Accordingly, postharvest processing strategies that induce metabolic modification have gained increasing attention as practical approaches to promote the conversion of glycosylated forms into their corresponding aglycones, thereby increasing the relative abundance of bioactive aglycone metabolites in plant-derived materials.^19^

Accordingly, postharvest processing strategies that induce metabolic modification have gained attention as practical approaches to enhance the functional potential of plant-derived materials.^20^ Among these strategies, lactic acid bacteria (LAB)-based fermentation is widely used in food processing and is known to remodel secondary metabolites through enzymatic hydrolysis and biotransformation. LAB play fundamental roles in industrial fermentation, producing organic acids, amines, vitamins, and bacteriocins while fermenting carbohydrates to lactic acid.^21^ Numerous LAB strains have been reported to enhance functional component content and associated biological activities in a variety of plant matrices, including soybean,^22^ berries,^23^ African nightshade,^24^ and avocado.^25^ Within LAB, the genus *Lactobacillus* encompasses more than 200 species, and their diverse genomic and biochemical characteristics necessitate careful strain selection.^26^

Therefore, this study aimed to investigate whether lactic acid bacteria-mediated fermentation could induce metabolic remodeling of *C. asiatica* through microbial biotransformation and thereby alter its secondary metabolite composition and associated bioactivity profiles relevant to Alzheimer’s disease-associated pathology.

## 2 Results and Discussion

### 2.1 Effects of Fermentation on Secondary Metabolites

To determine whether lactic acid bacteria (LAB) fermentation could remodel the secondary metabolite profile of *Centella asiatica* toward bioactive aglycone-dominant forms, nine LAB strains (presented in Figure 2) were applied, and the resulting changes in triterpenoid composition were systematically analyzed. Before the fermentation, raw *C. asiatica* (NF) material exhibited the following concentrations: 19.4 ± 0.5 mg/g of MS, 7.8 ± 0.1 mg/g of AS, 2.9 ± 0.1 mg/g of MA, and 4.1 ± 0.0 mg/g of AA. Postfermentation, the transformation in composition became evident, as depicted in Figure 3 (chromatogram suggested in Figure S1). This transformation indicated a decrease in glycosidic forms and an increase in acidic forms. Quantitatively, the fermentation process led to a significant enhancement in aglycones, with MA levels increasing by 8.0–12.0 times and AA levels by 4.8–7.3 times. Notably, the highest concentrations of AA (29.8 ± 0.2 mg/g) and MA (34.8 ± 0.5 mg/g) were observed in L5, as presented in Figure 3.

**Figure 2:**
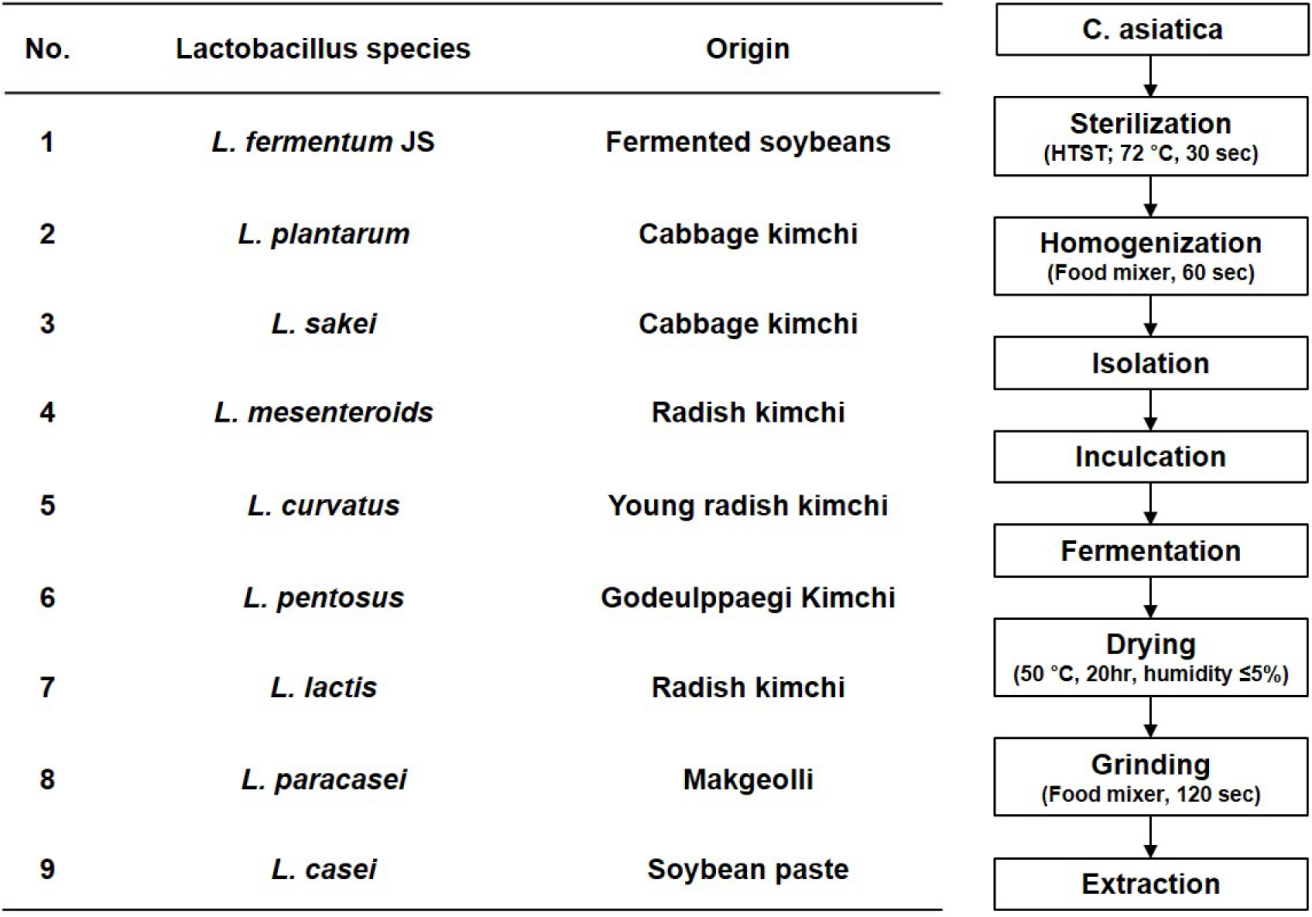
Types of *Lactobacillus* species used in this study and a schematic representation of the fermentation process.

**Figure 3:**
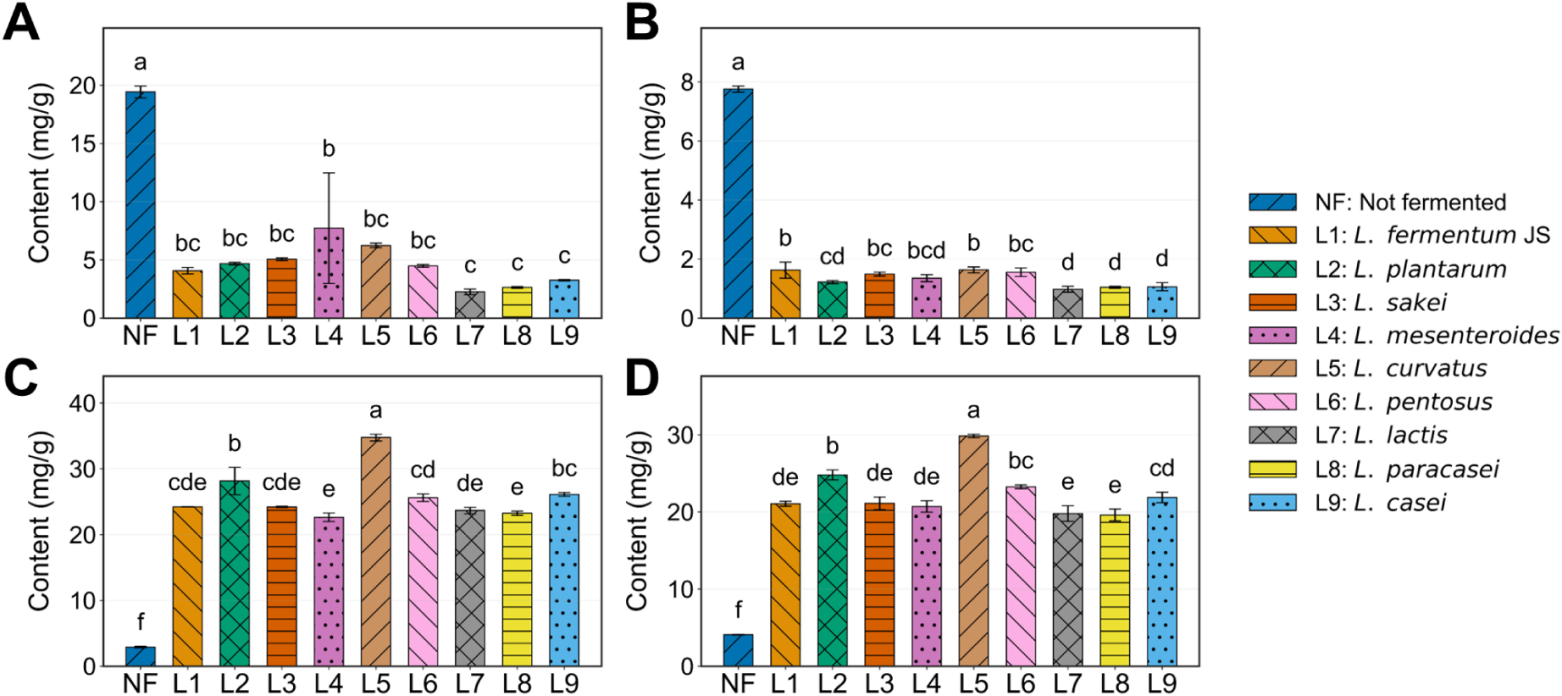
Quantitative results of four main centellosides, including madecassoside (A; MS), asiaticoside (B; AS), madecassic acid (C; MA), and asiatic acid (D; AA), in nonfermented (NF) and *C. asiatica*-fermented (CAF) extracts. Data are expressed as the mean of triplicate samples ± standard deviation (SD). Bars sharing at least one letter (e.g., “ab”) are not statistically significantly different according to Tukey’s HSD method at a significance level of *p* < 0.05.

To provide mechanistic context for the observed glycoside-to-aglycone conversion, fermentation is a metabolic process harnessing LAB, encompassing genera such as *Pedicoccus*, *Lactobasillus*, *Streptococcus*, *Latococcus*, *Leuconostoc*, among others.^27^ Microbial enzymes, including β-glucosidase, chitinase, xylanase, tannase, esterase, and lipase, can convert these compounds into aglycones through hydrolysis.^19^ In particular, β-glucosidase contributes to the conversion of flavonoid conjugates and bound phenols into aglycones and free phenols. These bioconversions can result in increased bioavailability of polyphenols as well as changes in their pharmacological properties.^28^ Numerous studies have explored fermentation to convert glycosidic forms into aglycone forms, with ginseng fermentation being one of the most notable examples. LAB from kimchi produce hydrolytic enzymes, and the β-glucosidase activity of these bacteria has been reported to be among the glycosyl hydrolases responsible for the bioconversion of ginsenoside Rb1 into Rg3 and Rg5.^29^ In this context, the apparent postfermentation increase in the summed contents of glycosylated triterpenoids (MS + MA) and the corresponding acidic forms (AS + AA) may not necessarily reflect de novo biosynthesis by LAB. Fermentation can alter the plant matrix and thereby improve the release, extractability, and analytical recovery of compounds already present in the raw material; similar increases in measured concentration attributable to enhanced extraction efficiency during LAB fermentation have been reported.^30^ Accordingly, we interpret these trends as being more consistent with compositional shifts from glycosylated forms toward their corresponding aglycones than with net metabolite production. Taken together, these results indicate that LAB fermentation primarily drives a structural shift in the triterpenoid profile of *C. asiatica*, characterized by the conversion of glycosylated triterpenoids into their corresponding aglycone forms rather than a simple increase in total metabolite content.

Fermentation of *C. asiatica* effectively induced the conversion of glycosidic forms (MS and AS) into their corresponding acidic forms (MA and AA). In contrast, *in vitro* assays combined with HPLC profiling revealed that fermentation exerted a negative effect on the phenolic and flavonoid contents. As shown in Figure 4, samples fermented with the L5 strain exhibited a 0.36-fold and 0.26-fold reduction in total phenolic and total flavonoid contents, compared with the NF control, consistent with a previous study. Previous studies have shown that flavonoids, particularly certain dihydrochalcones such as aspalathin and nothofagin, can undergo enzymatic and chemical oxidation during fermentation, leading to their conversion into other polyphenol derivatives.^31^ Methanolic extracts of *C. asiatica* have been reported to contain catechin, epicatechin, quercetin, myricetin, kaempferol, rutin, and naringenin.^32^ And most of these phenolic compounds, including gallic acid, catechin, chlorogenic acid, rutin, luteolin, rosmarinic acid, quercetin, and kaempferol, have been shown to decrease following fermentation.^33^ Collectively, these results demonstrate that LAB fermentation induces differential changes across metabolite classes, characterized by a reduction in phenolic and flavonoid contents alongside a pronounced increase in the contents of triterpenoid aglycones. Among the tested strains, L5 was identified as the most effective in enhancing the levels of MA and AA. Although the present study primarily addressed secondary metabolite remodeling, it is reasonable to expect that primary metabolites, including soluble sugars, organic acids, and amino acids, may also be modulated during fermentation through substrate utilization and organic acid production by LAB.^21^ Systematic profiling of the primary metabolite pool would provide further insight into fermentation-driven metabolic reorganization and represent an important direction for future studies.

**Figure 4:**
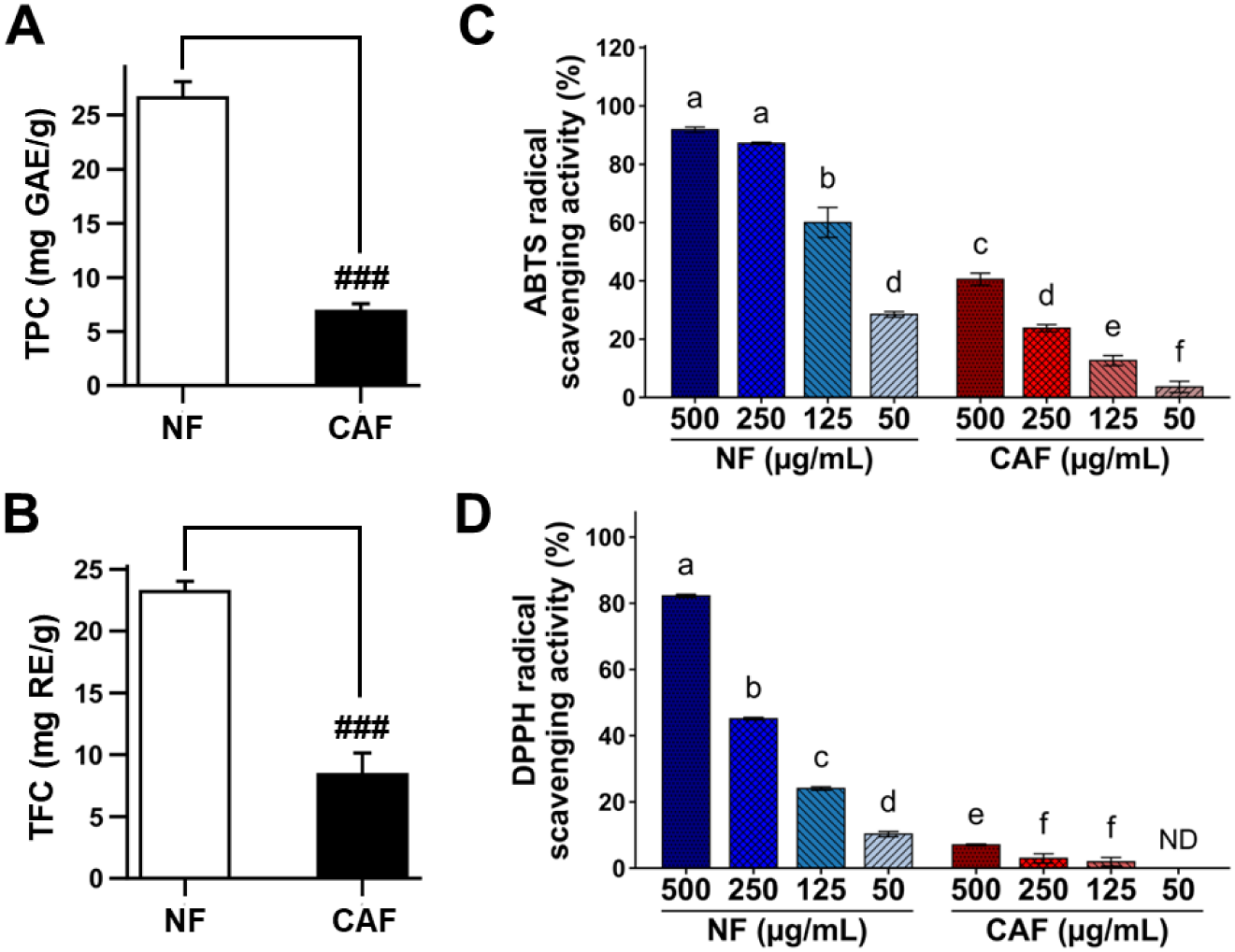
(A) Total phenolic content and (B) total flavonoid content were measured in samples fermented with the L5 strain using in vitro assays with Folin–Ciocalteu’s phenol reagent and the aluminum chloride colorimetric assay, respectively. Antioxidant activities were evaluated based on (C) ABTS radical scavenging activity and (D) DPPH radical scavenging activity. Data are presented as the mean ± standard deviation. In TPC and TFC analyses, Student’s *t*-test results are indicated using symbols (###: *p* < 0.001), while ANOVA followed by Tukey’s test was applied to analyze antioxidant activity results. Same letter is not statistically significant according to Tukey’s HSD method at a significance level of *p* < 0.05. NF, nonfermented extract; CAF, *C. asiatica*-fermented extract; TPC, total phenolic content; TFC, total flavonoid content; ABTS, 2,2′-azinobis­(3-ethylbenzothiazoline-6-sulfonic acid); DPPH, 2,2-diphenyl-1-picrylhydrazyl.

Importantly, the strain-specific differences observed in this study indicate that microbial selection plays a critical role in directing metabolic conversion efficiency, particularly in enhancing triterpenoid aglycones such as MA and AA.

### 2.2 Antioxidant and Anti-inflammatory Activities of Fermented *C. asiatica*

Additionally, the DPPH radical scavenging activity and ABTS radical scavenging activities demonstrated two highlighted results. First, *C. asiatica* exhibited stronger activity in ABTS radical scavenging activity compared to the DPPH radical scavenging activity. Second, NF showed significantly stronger antioxidant activities compared to the fermented *C. asiatica* using L5 (CAF) (Figure 4C and D). However, interestingly, the increased content of MA and AA through fermentation led to enhanced anti-inflammatory activity (Figure 5). The effect of standards on cell viability was assessed using the MTT assay after treatment with LPS and varying concentrations (10–40 μg/mL) of NF and CAF. These concentrations demonstrated that cell viability remained largely unaffected up to 40 μg/mL, with over 90% cell viability observed. Investigation of the effects of NF and CAF samples on LPS-induced NO production in RAW 264.7 cells was assessed by Griess assays, and NO content was calculated from a calibration curve (Y = 0.0048X + 0.0018, R^2^ = 0.9997), produced by nitrate (0–50 μM). The results showed that the amount of NO released by RAW 264.7 cells was significantly stimulated by LPS treatment (Figure 5B). Both NF and CAF showed no cytotoxicity at concentrations up to 40 μg/mL. Notably, CAF demonstrated a 1.9-fold enhancement in NO inhibitory activity relative to NF at this concentration. Together, these findings indicate that the fermentation-induced increase in MA and AA is associated with enhanced anti-inflammatory activity, even though overall antioxidant capacity was reduced.

**Figure 5:**
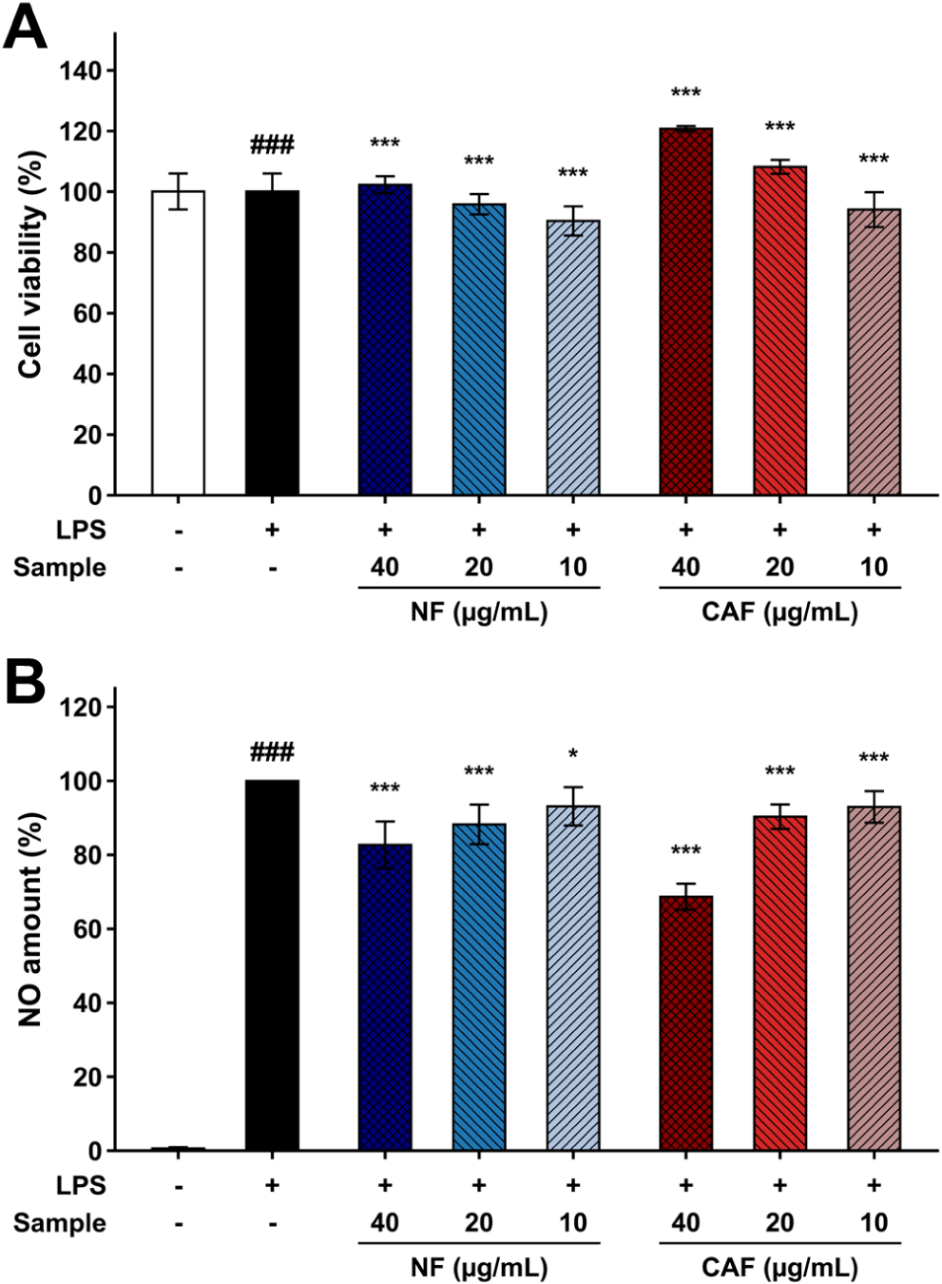
(A) Cell viability and (B) NO amount of the LPS-induced macrophage RAW 264.7 cells pretreated with NF and CAF samples. The data represent the mean ± standard deviation (SD) of three independent experiments. The symbol ### indicates *p* < 0.0001 between the LPS-treated and LPS-untreated groups. The symbols *, **, and *** indicate *p* < 0.05, 0.01, and 0.005, respectively, between sample-treated groups and the LPS-treated control.

As shown in Figure 6A, the cumulative proportion of variance explained by the first two principal components (PC1 and PC2) accounts for 99% (96% by PC1 and 3% by PC2) of the variance in the data. Through PCA, it was revealed that the secondary metabolites were also influenced along with biological activities. For discrimination, two perpendicular lines representing the double filtering criteria were set with a *p*-value <0.05 and |log_2_(FC)| > 2.0 in the volcano plot.^34^ All the variables were identified as important discriminant factors with high log_2_(FC) values (Figure 6B) and their normalized strip plots were presented in Figure 6C. This distinct difference between NF and CAF also influenced the correlation analysis, affecting the relationships among variables. In both NF and CAF, variables that showed high levels exhibited a very strong correlation (r values >0.9) (Figure 6D). Consistent with these observations, multivariate analyses (PCA and correlation analysis) revealed that fermentation-induced metabolite remodeling was accompanied by coordinated shifts in biological activity parameters rather than isolated changes in individual metabolites.

**Figure 6:**
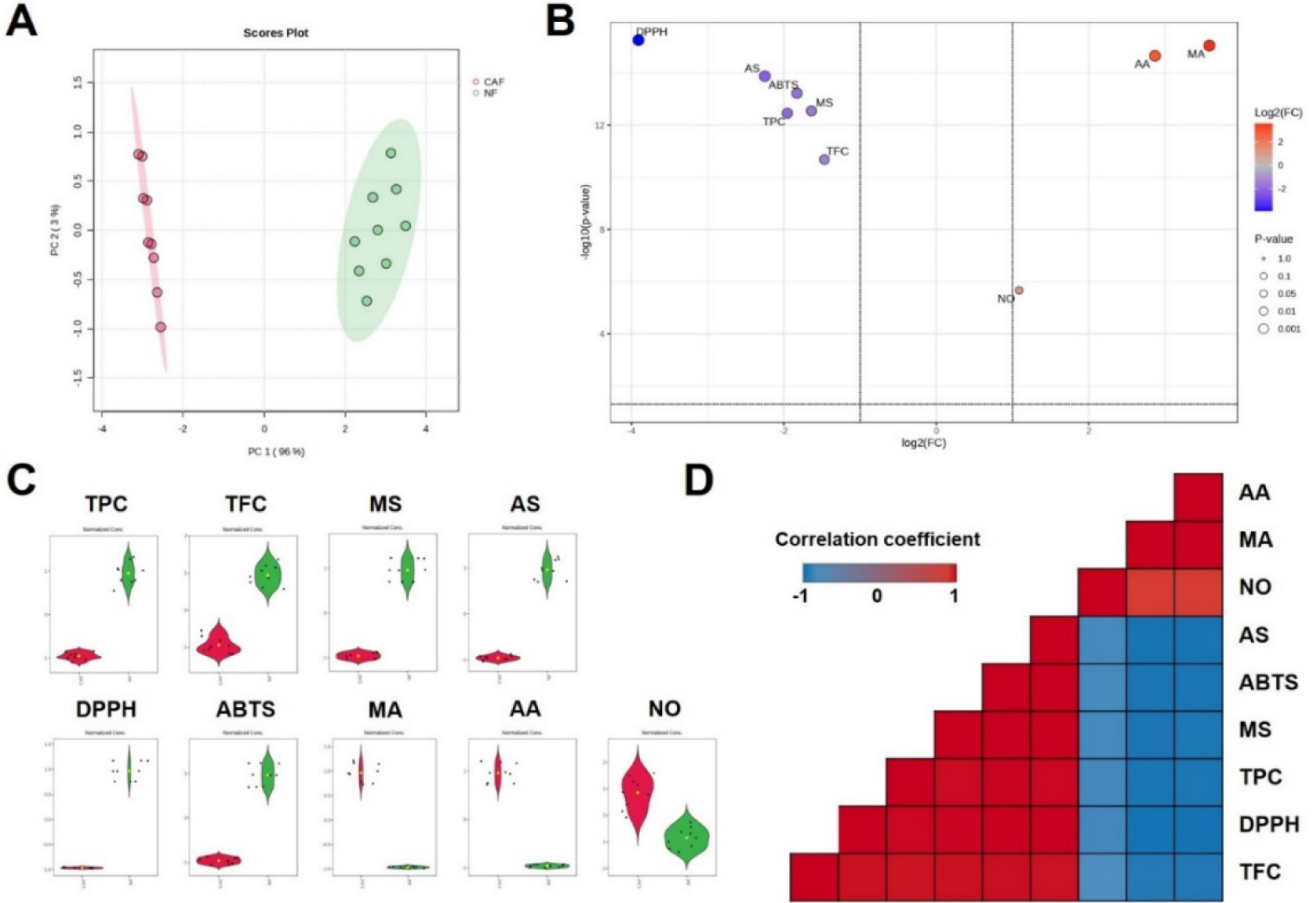
(A) PCA scores plot and (B) volcano plot were generated for the comparative analysis between nonfermented (NF) and fermented (CAF) *C. asiatica*. (C) Strip plots display variables related to secondary metabolites (TPC, TFC, MS, AS, MA, and AA) and biological activities associated with the early stages of Alzheimer’s disease (DPPH, ABTS, and NO). (D) Pearson’s correlation analysis between variables was also conducted. In the scores plot and strip plots, plot and bar colors represent groups (green: NF, red: CAF). In the volcano plot, colors indicate up- and downregulated variables (blue: higher in NF; red: higher in CAF). In the correlation analysis, the correlation coefficient (r) is visualized using box colors (red: 1, blue: −1).

The biological activities of *C. asiatica* are strongly influenced by the chemical nature of its constituent metabolites. Phenolic compounds are widely recognized as primary contributors to antioxidant activity due to their redox properties and structural diversity, which determine their radical-scavenging capacity and solubility.^35^ Accordingly, the observed reduction in the total phenolic content following fermentation is consistent with the decreased antioxidant capacity measured in DPPH and ABTS assays. Previous studies have suggested that fermentation can induce the degradation or transformation of phenolic compounds into other derivatives or byproducts through enzymatic and oxidative reactions, thereby reducing their overall antioxidant potential.^36^ In contrast, centellosides represent a distinct class of bioactive triterpenoids whose pharmacological properties are highly dependent on their structural form. While the glycosylated triterpenoids MS and AS have been primarily associated with wound-healing activity, their corresponding acidic aglycones, MA and AA, have been reported to exert more pronounced anti-inflammatory effects.^15,37^ These structural differences underscore the divergent functional roles of phenolic compounds and triterpenoid aglycones in determining the biological outcomes of *C. asiatica* fermentation.

Aging-associated oxidative stress is widely recognized as a critical initiating factor in AD, contributing to early molecular damage through increased oxidation of DNA, RNA, lipids, and proteins.^38^ Such oxidative insults often precede the formation of amyloid-β plaques and tau-based neurofibrillary tangles.^39^ Moreover, Aβ oligomers can further amplify oxidative stress by generating free radicals during aggregation, thereby promoting the formation of oligomers and protofibrils and inducing memory dysfunction.^40^ In response to this elevated level of oxidative stress, microglia mediate immune responses in neurodegenerative diseases, including AD. Proinflammatory microglia generated by oxidative stress are closely associated with protein aggregate pathologies. Therefore, increased oxidative stress and activated inflammatory mechanisms are closely linked to AD.^41^ Consequently, while antioxidant defenses may play a protective role during the early stages of Alzheimer’s disease, anti-inflammatory mechanisms are increasingly recognized as more directly involved in limiting disease progression. In this context, therapeutic strategies targeting inflammatory pathways have gained attention as promising approaches for Alzheimer’s disease prevention and management.^42^

Based on the antioxidant and anti-inflammatory assays presented above, taken together, the results of this section suggest a functional reorganization of bioactive constituents following LAB fermentation of *C. asiatica*. Glycosylated triterpenoids and phenolic compounds, which are more closely associated with antioxidant activity, appear to contribute primarily to early-stage oxidative defense. In contrast, fermentation-enhanced acidic triterpenoids, particularly MA and AA, are more strongly linked to anti-inflammatory activity, which is considered a key determinant of disease progression in Alzheimer’s disease. This stage-dependent complementarity highlights the potential of fermentation-induced metabolic remodeling as a strategy to shift the functional emphasis of *C. asiatica* toward inflammation-oriented preventive mechanisms relevant to neurodegenerative disorders.

### 2.3 Anti-Alzheimer’s Activity of Fermented *C. asiatica*

To investigate the anti-inflammatory-based anti-Alzheimer’s effects of CAF in the brain, a transgenic mouse model of AD, Tg-AD, was employed. In the brain, microglia and astrocytes are predominant glial cells that contribute to immune surveillance and the maintenance of neural homeostasis. These cells produce numerous chemokines and cytokines that are involved in inflammatory responses associated with neurodegenerative diseases.^43^ Alzheimer’s patients exhibit elevated levels of these proinflammatory cytokines, including tumor necrosis factor (TNF) and interleukins (ILs).^44,45^ Among these cytokines, IL-1β is a key proinflammatory cytokine produced by microglial cells surrounding Aβ plaques.^44^ In addition, Aβ itself induces TNF-α production in microglial cells. Consequently, upregulated TNF-α and IL-1β promote amyloid precursor protein synthesis and tau protein hyperphosphorylation through the activation of mitogen-activated protein kinase signaling pathways.^43^ In this study, Tg-AD mice exhibited significantly increased expression levels of TNF-α and IL-1β, whereas CAF treatment markedly suppressed these proinflammatory cytokines (Figure 7A and B). These results indicate that CAF effectively attenuates neuroinflammatory responses in the AD brain, suggesting its potential to modulate microglia-mediated inflammatory signaling associated with AD pathology.

**Figure 7:**
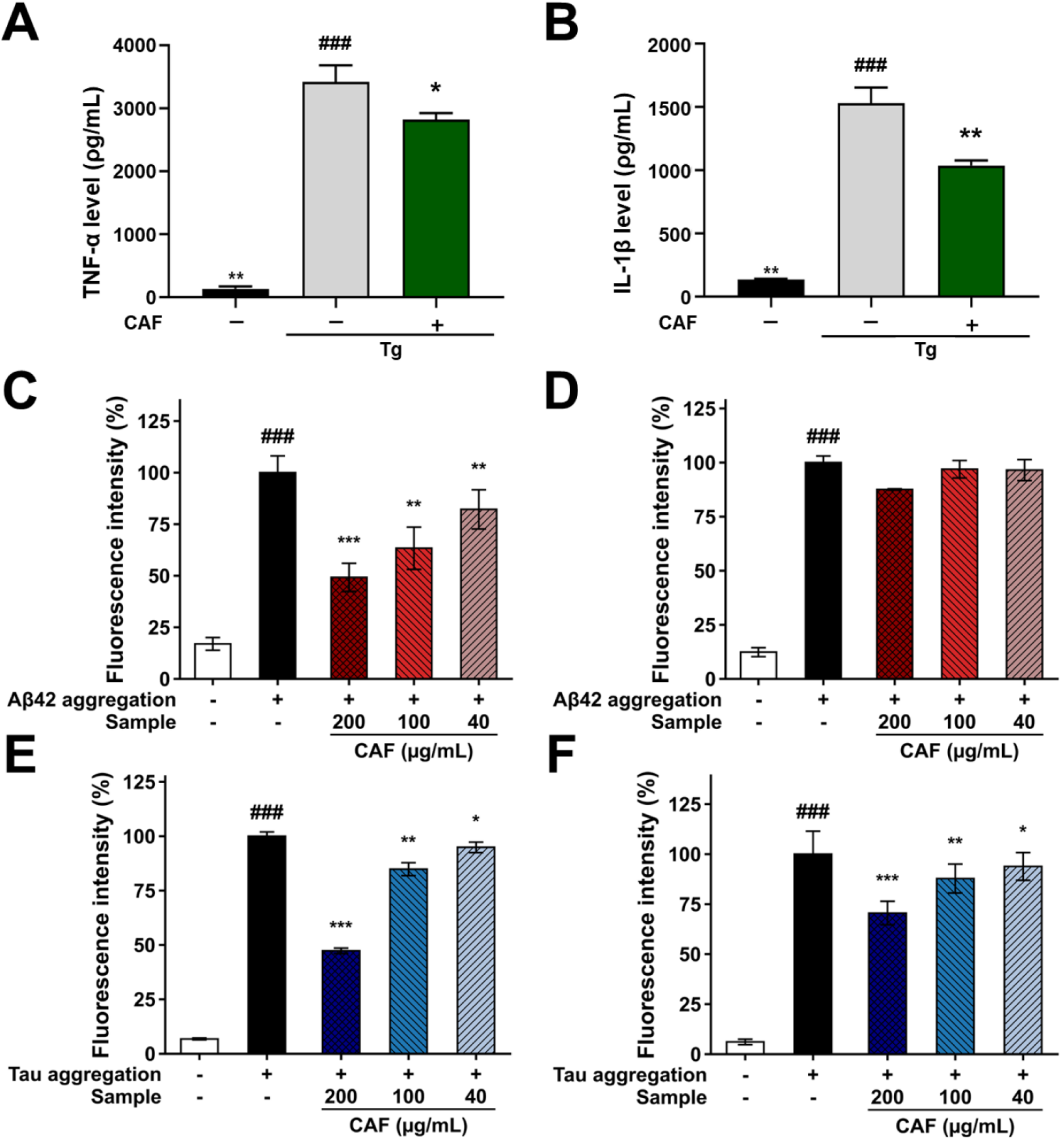
Predominant proinflammatory cytokines in the central nervous system, including (A) TNF-α and (B) IL-1β, were measured using an ELISA kit. Additionally, the inhibitory activity against the fibril formation and disassembly of oligomeric forms into monomeric forms of Aβ (C and D) and tau proteins (E and F), which are representative diagnostic biomarkers of Alzheimer’s disease, was evaluated.

In parallel with its antineuroinflammatory effects, CAF also exhibited anti-Alzheimer’s activity through the modulation of amyloidogenic and tau protein-related processes. The inhibition of Aβ and tau protein aggregation, as well as the promotion of their disaggregation, was evaluated and is presented in Figure 7. The aggregation of synthetic Aβ and tau peptides was confirmed by a significant increase in fluorescence intensity (*p* < 0.0001). CAF significantly and dose-dependently reduced Aβ fibril formation to levels comparable to those of monomeric Aβ (Figure 7C). Furthermore, CAF promoted the disassembly of oligomeric Aβ into monomeric species in a dose-dependent manner (Figure 7D). A similar trend was observed in tau protein aggregation. CAF treatment reduced tau aggregation levels (Figure 7E), and thioflavin T fluorescence intensity was also markedly decreased when CAF was incubated with preformed tau K18 aggregates (Figure 7F). Collectively, these results demonstrate that CAF effectively inhibits aggregation and promotes the disaggregation of both Aβ and tau proteins. The simultaneous suppression of protein aggregation and enhancement of disaggregation suggest that CAF interferes with amyloidogenic and tau-associated pathological processes that are central to Alzheimer’s disease progression.

### 2.4 Animal Test

#### 2.4.1 Reduction of Amyloid Plaque Burden in APP/PS1 Transgenic Mice by CAF

Based on the observed inhibitory and disaggregating effects of CAF on Aβ and tau aggregation in vitro, we examined whether CAF could attenuate Alzheimer’s disease-related pathological features in an animal model. Consequently, CAF was administered to APPswe/PSEN1dE9 double-transgenic mice (APP/PS1), a well-established model of Alzheimer’s disease. This model overexpresses amyloid precursor protein (APP) and presenilin-1 (PS1), leading to early Aβ plaque deposition and progressive cognitive impairment.^46^ Notably, APP/PS1 mice exhibit increased Aβ plaque formation from approximately 4–5 months of age, with measurable cognitive deficits emerging around 6–8 months.^47^ The effects of CAF on amyloid-related neuropathology were evaluated by using immunohistochemistry and thioflavin S (ThS) staining. Histological analysis revealed a marked reduction in Aβ plaque burden in the CAF-treated group compared to the vehicle-treated controls. This observation was further supported by quantitative analysis of Aβ plaque numbers (Figure 8).

**Figure 8:**
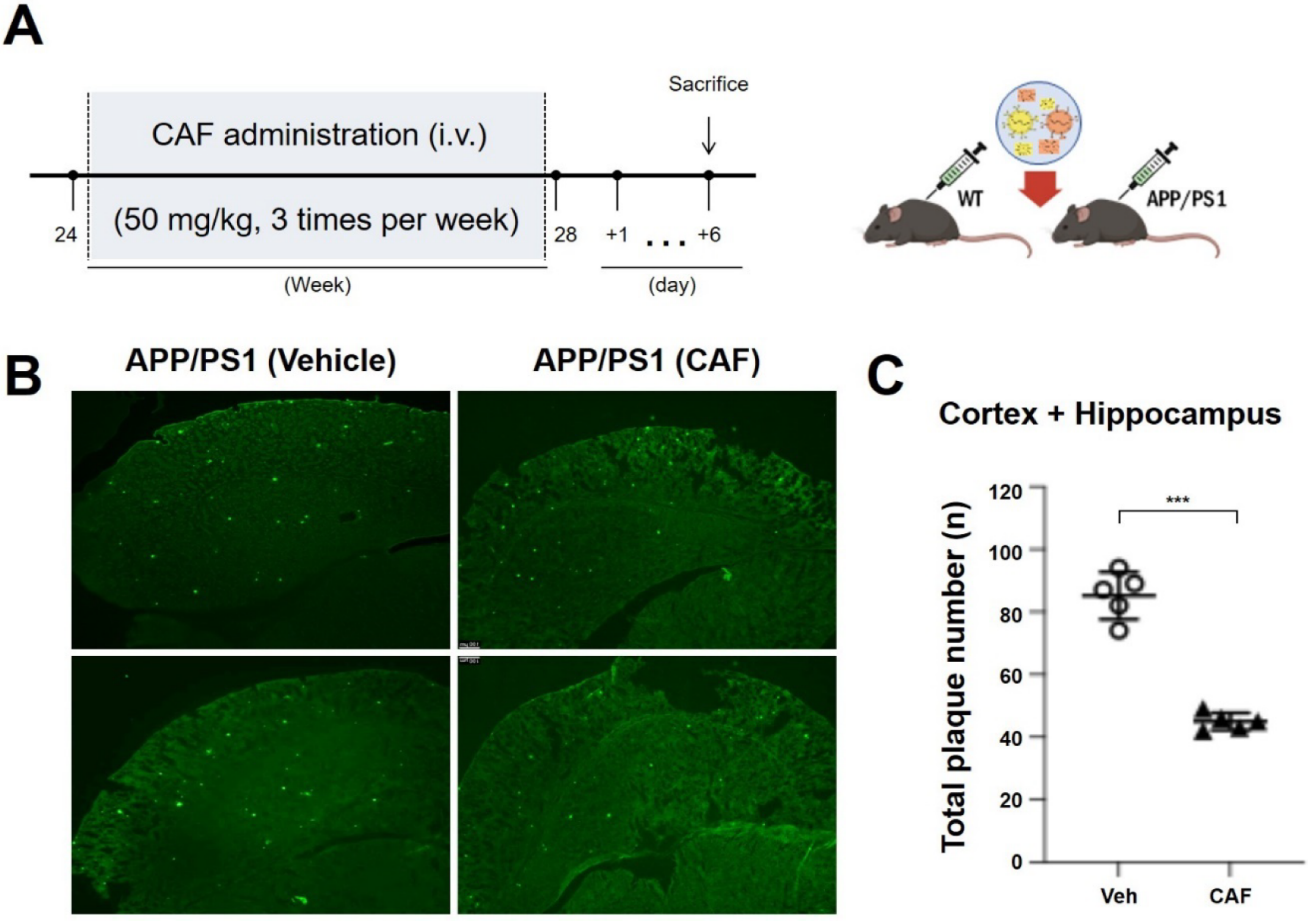
(A) Timeline of the treatments. Mice were administered orally with vehicle (Veh) or CAF (50 mg/kg) for 1 month. (B) In vivo detection of amyloid plaques in a mouse model of Alzheimer’s disease using ThS staining. And (C) the total plaque number was counted. *** indicates the significant difference (*p* < 0.005) between Veh and CAF treatment.

These in vivo observations are consistent with the antiamyloid aggregation effects observed in vitro and suggest that fermentation-induced metabolic remodeling may modulate amyloid-related markers in this model. Collectively, the results underscore the role of lactic acid bacteria-mediated biotransformation in reshaping the biological attributes of plant-derived triterpenoids and provide insight into how metabolic forms of these compounds are linked to processes relevant to Alzheimer’s disease-associated pathology.

## 3 Conclusions

Fermentation is increasingly recognized as an effective approach for modulating the chemical composition of plant-derived materials through metabolic transformation rather than the simple enrichment of individual constituents. In this study, lactic acid bacteria-mediated fermentation of *Centella asiatica* (L.) Urb. induced pronounced remodeling of secondary metabolites, notably characterized by the enrichment of acidic triterpenoids and a concomitant reduction in glycosylated triterpenoids and phenolic compounds. This metabolic shift was accompanied by corresponding changes in bioactivity profiles, including reduced antioxidant capacity but enhanced anti-inflammatory activity and modulation of the amyloid plaque burden in the APP/PS1 model. These findings illustrate how microbial biotransformation can reshape the biological properties of plant-derived triterpenoids by altering their metabolic forms, thereby potentially influencing biological responses associated with neuro-inflammation and protein aggregation. Overall, this work underscores fermentation-driven metabolic remodeling as a useful strategy for investigating structure–activity relationships in natural products and for understanding how the chemical form contributes to bioactivity in complex biological contexts.

## 4 Methods

### 4.1 Plant Material

*C. asiatica* used in this experiment were cultivated using a vertical farming system at the Korea Institute of Science and Technology (Gangneung, Korea). For cultivation, *C. asiatica* plants were exposed to fluorescent lights, with a set light–dark cycle of 14/10 h. Throughout the cultivation period, temperatures were maintained at 25 °C during the day and 18 °C during the night, while the humidity was consistently kept at 70 ± 5%. Following an 8-week cultivation period, the *C. asiatica* plants were harvested and subjected to drying using a cool-air dryer at a temperature of 30 ± 2 °C. Subsequently, these dried samples were used for analysis and fermentation.

### 4.2 Fermentation Process

#### 4.2.1 Chemicals and Culture Media

Slightly acidic electrolyzed water (Shinsunglab Medical Co., Ltd., Gangneung, Gangwon, Republic of Korea), De Man, Rogosa, and Sharpe Broth (MRS broth), and Agar (BD Difco, Sparks, MD, USA) were purchased, and all of the reagents were of analytical quality.

#### 4.2.2 Bacterial Strains, Growth Conditions, and Preparation

LAB strains used in this study were acquired from Well-being LS Co., Ltd., Gangneung, Gangwon, Republic of Korea (Figure 2). To isolate LAB strains, various isolate resources were collected, and isolation was accomplished through the following process: 1) plate spreading and incubation, 2) repeated streaking, 3) liquid culture, 4) verification of single colony isolation, and 5) base sequence confirmation.

The strains were maintained in glycerol stock; the subculturing was performed using MRS broth; and the bacterial strains were grown at 37 ± 2 °C for 24 h. Further, the bacterial samples were centrifuged at 8,000 rpm for 20 min, and the pellet was dried and stored at 4 ± 2 °C until use. Prior to fermentation, the strains were subcultured once.

#### 4.2.3 Preparation of Fermentation

The fermentation process is described in Figure 2. First, *C. asiatica* was soaked in slightly acidic electrolyzed water in a 1:2 ratio at 20–25 °C for 10 min. After removing the water, the samples were blanched in 75 ± 2 °C water in a 1:2 ratio for 30 s, followed by rinsing in 20–25 °C water. After sterilization, the samples were dried using a hot air dryer (UDS-6533F, KyungDong Navien Co., Ltd., Seoul, Republic of Korea) at 50 °C for 8 h, ground using an electric crusher into a fine powder, and separated via mesh (279–381 μm). *C. asiatica* powder was soaked in sterile distilled water (1:4 ratio) and inoculated with LAB powder (1.0 × 10^7 cfu/g, 10% of total weight), and the tray fermentation method was performed for 22 h at 37 °C. After fermentation, fermented *C. asiatica* was again dried using a hot air dryer at 50 °C for 8 h. After drying, it was ground into a fine powder and sieved to 279–381 μm

### 4.3 Quantitative Analysis of Four Centellosides

#### 4.3.1 Preparation of Extracts

Samples both with or without fermentation underwent the extraction process. Samples were extracted with methanol (1 g per 50 mL) using an ultrasound sonicator at 55 °C for 90 min. After the extraction, samples were centrifuged at 13,000 rpm for 10 min and the supernatant was filtered through a 0.45 μm membrane filter (Whatman). And the gathered extracts were clearly concentrated by nitrogen evaporation and dissolved in DMSO to a concentration of 40 mg/mL.

#### 4.3.2 Analysis of Triterpenoids

For the analysis, samples were analyzed using a UPLC-PDA instrument (Nexera X3; Shimadzu, Kyoto, Japan) coupled with a system controller (SCL-40), an autosampler (SIL-40C X3), a binary pump (LC-40B X3), an oven (CTO-40C), and a PDA (SPD-M40). Samples (1 μL), incubated in the autosampler at 20 °C, were injected and loaded into a YMC C18 Triart column (100 × 2.1 mm ID, 1.9 μm; YMC, Japan) conditioned at 35 °C. The two organic solvents (A: water; B: acetonitrile) were flowed as mobile phases with a flow rate of 0.35 mL/min and a gradient system was applied to isolate compounds. The initial ratio of solvent B was 23% and was maintained for 2 min. Subsequently, the B ratio was consistently increased to 23–35% at 2–7 min, 35–50% at 7–12 min, 50–75% at 12–18 min, and 75–100% at 18–27 min. Starting from 27 min, the B ratio was set to 100% and held for 4 min for column washing, and from 33 to 37 min, the column was re-equilibrated with the initial solvent system.

### 4.4 Total Phenolic and Total Flavonoid Content

The total phenolic content (TPC) and total flavonoid content (TFC) were measured by colorimetric assays.^48^ First, TPC was determined using the Folin–Ciocalteu method. Briefly, samples (10 mg/mL, 20 μL) were mixed with 200 μL of 2% Na_2_CO_3_. After 3 min, 20 μL of 1 N Folin–Ciocalteu reagent was added, and the mixture was incubated at room temperature for 30 min. After the incubation, absorbances converting from yellow to blue were recorded at 750 nm and TPC was expressed as gallic acid equivalents (mg GAE/g) with calibration curve (Y = 0.0029X + 0.0229; R^2^ = 0.9955).

As for TFC, it was measured by an aluminum chloride colorimetric assay with some modifications. In the 96-well plate, 50 μL was taken for each sample (10 mg/mL) and 130 μL of methanol was added followed by the immediate addition of 20 μL of 1 M potassium solution. After 5 min, 20 μL of 10% AlCl_3_ solution was added and incubated for 10 min at room temperature. Then, the absorbances were measured at 415 nm and expressed as rutin equivalents (mg RE/g) with a calibration curve (Y = 0.0018X – 0.0524; R^2^ = 0.9976).

### 4.5 Antioxidant Activity

To evaluate the antioxidant properties, DPPH radical scavenging activity and ABTS radical scavenging activity were performed^49^ with few modifications. DPPH (2,2-diphenyl-1-picrylhydrazyl) is a stable free radical typically measured in organic solvent at 517 nm, whereas ABTS (2,2′-azinobis­(3-ethylbenzothiazoline-6-sulfonic acid)) quantifies the decolorization of the preformed ABTS•+ radical cation and is applicable in both aqueous and organic systems (734 nm).^50^ Prior to the experiment, DPPH powder was dissolved in methanol to achieve an absorbance of 1.0 (±0.05) at 517 nm. And ABTS tablet (10 mg) was dissolved in water, 2.45 mM K_2_S_2_O_8_ was added, and the mixture was left to stand in the dark for 16 h to generate ABTS radical cations. It was diluted with ethanol to an absorbance of 0.7 (±0.02) at 734 nm.

Briefly, in the 96-well plate, 10 μL of diverse concentrations of samples (50–500 μg/mL) was added, and the mixture was reacted with 190 μL of each reagent. For the reaction, DPPH radical scavenging activity was incubated for 30 min, and ABTS radical scavenging activity was incubated for 10 min at room temperature under dark conditions. Absorbances were read, and the percentage of activity was calculated following the reference paper.

### 4.6 Anti-inflammatory Activity

#### 4.6.1 Cell Incubation

RAW 264.7 cells (mouse macrophage cell line) were provided by the Korea Cell Line Bank (KCLB, Seoul, Korea) and used for anti-inflammatory activity. RAW 264.7 cells were cultured in Dulbecco’s phosphate-buffered saline (DPBS) medium containing fetal bovine serum (FBS; U/mL) and antibiotics (U/mL penicillin and U/mL streptomycin) at 37 °C in a humidified 5% CO_2_.

#### 4.6.2 Cell Toxicity

Cell toxicity was assessed by the 2,5-diphenyl-2H-tetrazolium bromide (MTT) assay. RAW 264.7 cells were seeded on a 96-well plate with a density of 5 × 10^4^ cells per well and incubated for 24 h in a 5% CO_2_ incubator at 37 °C. After that, the old medium was removed and the plate was rinsed with DPBS. EZ-Cytoz solution was added into the well and incubated for 0.5–1 h in a 5% CO_2_ incubator at 37 °C, and the absorbance was measured at 450 nm by spectrophotometry.

#### 4.6.3 NO Assay

NO inhibitory activity was evaluated by the Griess reagent assay with some modifications. RAW 264.7 cells were seeded on a 96-well plate at a density of 5 × 10^4^ cells per well and incubated for 24 h in a 5% CO_2_ incubator at 37 °C. Fresh media with 50 μL of various concentrations of standards and samples were added to each well, and after 1 h, 50 μL of medium with or without 1 μg/mL of lipopolysaccharide (LPS) was added and incubated for another 24 h. After that, the culture supernatant was transferred to a new plate, and the NO concentration was detected using Griess reagent (1:1 ratio) by spectrophotometry at 540 nm.

### 4.7 Anti-Alzheimer’s Activity

#### 4.7.1 Aβ Preparation

Full-length Aβ (1–42) peptides were reliably synthesized using the Fmoc solid-phase peptide synthesis method^51^ and purification and analyzed using reverse-phase HPLC (1220; Agilent Technologies) and electrospray ionization mass spectrometry (ESI-MS) (6120; Agilent Technologies). Dimethyl sulfoxide (DMSO) was used for avoiding on-resin folding during the Fmoc solid-phase peptide synthesis. Purified Aβ (1–42) peptides were prepared as solutions (stock at 25 mM) by using DMSO and then diluted with deionized water.

#### 4.7.2 Tau Preparation

The DNA sequence encoding K18, a truncated form of human tau, was cloned and inserted into a pET vector. K18 mutants (K18-P301L, K18-C291S, and K18-C291S/C322S) were synthesized by using the QuikChange site-directed mutagenesis kit (Stratagene, San Diego, CA, USA). K18-P301L (35 μM) was purified and incubated in phosphate-buffered saline (PBS, pH 7.4) at 37 °C for 3–5 days. Subsequently, 0.1 mg/mL heparin (MW 18 kDa; Sigma, Livonia, MI, USA) and 100 μM DTT (Sigma) were added to induce tau aggregation.^52^

#### 4.7.3 Inhibition of Aβ Aggregation/Aβ Disaggregation Assays

The Aβ solutions were treated with samples (40, 100, and 200 μg/mL) and incubated for 5 days at 37 °C for the inhibition test. For the disaggregation test, Aβ solutions that were preaggregated by incubating at 37 °C for 5 days were mixed with standards and samples and incubated at 37 °C for another 5 days. Subsequently, the samples and ThT solutions were loaded onto 96-well black plates, and their fluorescence levels were measured immediately. The levels of Aβ-bound ThT were measured at 450 nm (excitation) and 485 nm (emission).

#### 4.7.4 Inhibition of Tau Aggregation/Tau Disaggregation Assays

For the inhibition test, samples (40, 100, and 200 μg/mL), K18 (a truncated form of human tau) in phosphate-buffered saline containing 0.1 mg/mL heparin, and 100 μM DTT were incubated at 37 °C for 3 days. For the disaggregation test, after K18 was stored in 0.1 mg/mL heparin and 100 μM DTT at 37 °C for 3 days, standards and samples were added to the aggregation mixture and incubated at 37 °C for 3 days. Subsequently, ThT was added to 96-well black opaque plates. The fluorescence levels of tau-protein-bound ThT were measured at 450 nm (excitation) and 485 nm (emission).

### 4.8 Neuroinflammatory Activity Measurement

Samples and standards were diluted in Reagent Diluent (1% BSA in PBS), added to each well at 100 μL per well, and incubated at room temperature for 2 h. The plasma samples were prepared by diluting 50 μL of plasma with 200 μL of 1% BSA in PBS (1:5 dilution). Standards were prepared by diluting the stock solution 1:100 to achieve 2000 pg/mL, followed by 1:2 dilutions in 1% BSA to obtain final concentrations of 2000, 1000, 500, 250, 125, 62.5, 31.3, and 0 pg/mL, and the wells were washed four times with 300 μL of PBS-T per well.

Detection antibody was prepared by diluting 1:100 in 1% BSA in PBS and added at 100 μL per well, incubated at room temperature for 2 h, and washed again four times with 300 μL of PBS-T. Streptavidin-HRP was prepared by diluting 1:40 in 1% BSA in PBS, added at 100 μL per well, incubated at RT for 20 min in the dark, and washed four times with 300 μL of PBS-T, and 100 μL of 1× TMB Substrate Solution was added to each well, and the color change was monitored for 5 to 10 min in the dark. When the desired color change was observed, 50 μL of Stop Solution (2N H_2_SO_4_) was added to each well, and the plate was gently tapped to mix the solution. Finally, the absorbance was measured at 450 nm by using a plate reader.

### 4.9 Biochemical Analysis of Brain Tissue in Alzheimer’s Disease Transgenic Mice

#### 4.9.1 Animals

All animal experiments were conducted using APP/PS1 double-transgenic mice [male, APP/PS1, B6.C3-Tg (APPswe, PSEN1dE9)] and wild-type (male, C57BL/6) mice. Both breeds were obtained from Jackson Laboratory (Bar Harbor, Maine, USA). The APP and PS1 genotypes were confirmed using PCR analysis with tail DNA, following the standard protocols of the Jackson Laboratory (Protocol Nos. 23370, 004462, and 31769). Both APP/PS1 and wild-type mice were housed in a laboratory animal breeding room at Dongguk University. The mice were maintained under controlled temperature conditions and were provided with food and water ad libitum. All animal experiments were performed in accordance with institutional guidelines and regulations and were approved by the Institutional Animal Care and Use Committee of Dongguk University (IACUC No. 2021-020-3).

#### 4.9.2 ThS Staining and Immunohistochemical Analyses

The mice were anesthetized with enflurane (5%) and euthanized. The brains extracted from wild-type and APP/PS1 mice were fixed in 4% paraformaldehyde (pH 7.4) and immersed in 30% sucrose for cryoprotection. The fixed brain samples were cut into 25 μm thick slices by using a Cryostat Microtome. For ThS staining, the sectioned brains were stained with 500 μM ThS dissolved in 50% ethanol for 7 min. Next, the sections were sequentially rinsed with 100%, 90%, and 50% ethanol and PBS at room temperature. Images were taken using a Leica DM2500 fluorescence microscope (Leica Microsystems).

#### 4.9.3 Statistical Analysis

Descriptive statistics were presented as mean ± standard deviation (n = 3). In terms of the NO inhibitory activity, whether there was a significant effect of LPS on NO production was indicated by the symbol (###) as well as significant aggregation. And ANOVA (Analysis of Variance) was performed to determine whether there was a difference between the groups in terms of the considered characteristics. Posthoc Tukey’s Honestly Significant Difference (HSD) test was used to determine significant differences. The statistical significance level was considered to be 5% (*p* < 0.05) and R-Studio was used for all statistical computations.

## Supplementary Material



## Data Availability

The raw numerical data sets underlying Figures 3, 4, 5, 7, and 8 are available from the corresponding authors upon reasonable request. Figure 6 presents integrated statistical analyses derived from these data sets and therefore does not have a separate raw data file.

## Notes

HPLC profiling chromatogram detected at 210 nm comparing nonfermented (NF) and fermented (CAF) samples (Figure S1) (PDF)

## ⊥

D.H.R.: Department of Plant Science, The Pennsylvania State University, University Park, Pennsylvania 16802, United States

## #

J.Y.C.: Department of Plant Pathology and Environmental Microbiology, The Pennsylvania State University, University Park, Pennsylvania 16802, United States.
