# Supplementary material for: Lactic Acid Bacteria-Mediated Fermentation Drives Metabolic Remodeling of Centella Asiatica (L.) Urb. toward Acidic Triterpenoids with Neuroinflammation-Related Bioactivity
Source: ACS Omega. 2026 May 4;11(19):28607–18. doi: 10.1021/acsomega.6c00935 (PMC13191678; doi:10.1021/acsomega.6c00935)
Supplement: Supplementary file 1 [file ao6c00935_si_001.pdf]

Lactic Acid Bacteria–Mediated Fermentation Drives Metabolic Remodeling of *Centella asiatica* (L.) Urb. toward Acidic Triterpenoids with Neuroinflammation-Related Bioactivity

Da Hye Ryu[1,‡,†], Jwa Yeong Cho[1,§,†], Jae Woon Jung[2†], Hyeong Ho Cha[1,3], Hye Min Kim[1,3], Na-Yun Park[1,3], Na-Hyun Ahn[2], Joo-Hee Lee[2], Jong Soon Park[4], Deuk-Sik Lee[4], Seung Hoon Yang[2,*], and Ho-Youn Kim[1,3,*]

[1] Smart Farm Research Center, Korea Institute of Science and Technology (KIST), Gangneung 25451, Republic of Korea

[2] Department of Biomedical Engineering, College of Life Science and Biotechnology, Dongguk University, Seoul, 04620, Republic of Korea

[3] Natural Product Applied Science, KIST School, University of Science and Technology (UST), Gangneung, Gangwon, 25451, Republic of Korea

[4] Life Science Institute, Well-being LS Ltd., Gangneung, Gangwon, 25451 Republic of Korea

† D.H.R., J.Y.C., and J.W.J. contributed equally to this work.

* Corresponding Authors
Email: shyang@dongguk.edu; hykim@kist.re.kr
Present Addresses
‡ D.H.R.: Department of Plant Science, The Pennsylvania State University, University Park, Pennsylvania 16802, United States.
§ J.Y.C.: Department of Plant Pathology and Environmental Microbiology, The Pennsylvania State University, University Park, Pennsylvania 16802, United States.
Author e-mails:

Number of tables: 0

Number of figures: 8

Number of supplementary data: 1 Figure

No.	Compound	Retention time (min)	Concentration range (μg/mL)	Calibration curve	Company	Cat. No	CAS. No
**1**	Madecassoside	5.9	20 – 1000	Y = 364.24X + 1045.8	SCBT	sc-279288	34540–22–2
**2**	Asiaticoside	7.1	20 – 1000	Y = 566.34X – 1401.8	chemcruz	sc-257101	16830-15-2
**3**	Madecassic acid	11.8	50 – 1000	Y = 1209.1X – 17178	chemcruz	sc-391157	18449-41-7
**4**	Asiatic acid	13.9	50 – 1000	Y = 1357.8X – 15314	Cayman	11818	464-92-6

Chromatographic profile detected at 210 nm

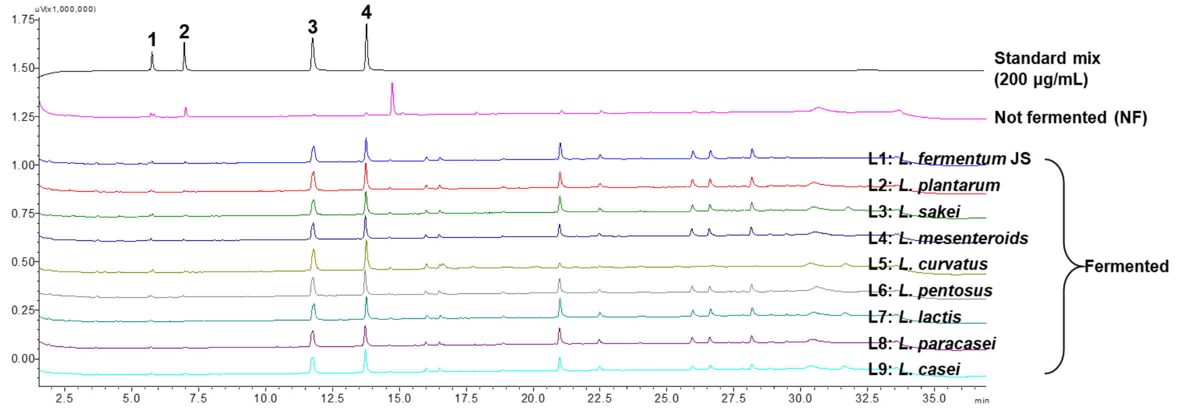

Figure S1. HPLC chromatographic profiles of non-fermented (NF) and fermented samples with diverse strains. The four authentic triterpenoid standards were analyzed under identical chromatographic conditions, and their retention times were used for peak assignment.
